# MiRNAs and LincRNAs: Could They Be Considered as Biomarkers in Colorectal Cancer?

**DOI:** 10.3390/ijms13010840

**Published:** 2012-01-16

**Authors:** Ruth Zarate, Valentina Boni, Eva Bandres, Jesús Garcia-Foncillas

**Affiliations:** 1 Laboratory of Pharmacogenomics, Division of Oncology, Center for Applied Medical Research (CIMA), University of Navarra, Pamplona-Navarra 31008, Spain; E-Mails: vboni@unav.es (V.B.); jgfoncillas@unav.es (J.G.-F.); 2 Laboratory of Clinical Genetics, University of Navarra, Pamplona 31008, Spain; 3 Laboratory of Immunology, Service of Hematology, Complejo Hospitalario de Navarra, Pamplona 31008, Spain; E-Mail: ebandres@unav.es; 4 Unit for the Research and Treatment of Gastrointestinal Malignancies, Department of Medical Oncology, University Clinic of Navarra (CUN), Pamplona-Navarra 31008, Spain

**Keywords:** biomarkers, cancer, prognostic, non-coding RNA

## Abstract

Recent advances in the field of RNA research have provided compelling evidence implicating microRNA (miRNA) and long non-coding RNA molecules in many diverse and substantial biological processes, including transcriptional and post-transcriptional regulation of gene expression, genomic imprinting, and modulation of protein activity. Thus, studies of non-coding RNA (ncRNA) may contribute to the discovery of possible biomarkers in human cancers. Considering that the response to chemotherapy can differ amongst individuals, researchers have begun to isolate and identify the genes responsible. Identification of targets of this ncRNA associated with cancer can suggest that networks of these linked to oncogenes or tumor suppressors play pivotal roles in cancer development. Moreover, these ncRNA are attractive drug targets since they may be differentially expressed in malignant *versus* normal cells and regulate expression of critical proteins in the cell. This review focuses on ncRNAs that are differently expressed in malignant tissue, and discusses some of challenges derived from their use as potential biomarkers of tumor properties.

## 1. Introduction

Use of predictive biomarkers should be an integral part of current clinical practice and be used as an aid to clinical experience and expertise in making patient therapy decisions.

Since the first miRNA was discovered [1,2] these have been added as a new level to gene regulation in normal as well as pathological cell function. It is estimated that more than 30% of the human genes are post-transcriptionally regulated by miRNAs [3].

In addition to well-described microRNAs, the growing knowledge of the mammalian non-coding transcriptome is revealing that the genome is also replete with highly conserved large ncRNAs (lncRNAs), which could have a major role in the development and progression of cancer [4], although their mechanisms of action remain less well understood [5].

Given the larger and growing focus on targeting RNAs for disease therapeutics, what we do know about the intrinsic biology of these small RNAs (miRNA) and long non-coding RNA (lncRNA) makes them potentially attractive targets for pharmacologic manipulation and prognosis markers.

The miRNA was referred initially as small temporal RNA (stRNAs) whith appearance during development [6]. Mature miRNAs regulate mRNA target degradation or translational repression by mediating sequence-specific targeting of its mRNA [7,8]. It is very important to consider that the number of miRNA binding sites can vary in the target mRNA affecting efficiency of translational repression [9].

In contrast to miRNAs, lncRNAs are mRNA-like transcripts ranging in length from 200 nt to ~100 kilobases (kb) lacking significant open reading frames. Many identified lncRNAs are transcribed by RNA polymerase II (RNA pol II) and are polyadenylated, but this is not a fast rule [10,11].

Large ncRNAs were first described during large scale sequencing of full-length cDNA libraries in the mouse [12]. One subclass of lncRNAs is called large or long intergenic ncRNAs (lincRNAs). These lincRNAs are exclusively intergenic and are marked by a chromatin signature indicative of transcription [5,13]. They show a clear evolutionary conservation that confirms the distinctive biological roles developed through diverse mechanisms [14–16].

Less than 2% of the total genome contains only 20,000 protein-coding genes whereas a substantial fraction of the human genome can be transcribed, yielding many short or lncRNAs with limited protein-coding capacity. Between them, lincRNA are mostly identified (>3000) but a few have been characterized (<1%).

Some lncRNAs are preferentially expressed in specific tissues [17]. But, compared to miRNA, nowadays few lincRNA has been characterized.

To hypothesize which miRNAs could be involved in translational regulation of candidate target even is necessary carried out an *in silico* analysis of putative miRNAs. Although, let-7 represents one of miRNA whose targets genes as RAS [18], HMGA2 [19], MYC [20], and p16^INK4A^ [21] were specifically validated and linked to its regulatory effect.

Features of malignant tumors, distinct from benign tumors, include invasion and metastasis. miR-21 is one of the most frequently upregulated miRNAs in cancers. In colorectal cancer (CRC), miR-21 promotes invasion, intravasation, and metastasis by downregulating *Pdcd4* [22].

We will focus on CRC carcinogenesis process and considering the recent up-date “the tumor hallmarkers” [23] describe the miRNAs and LincRNAs that have been implicated in this pathway. Given that a miRNAs regulate multiple targets and one target gene could be regulated by multiple miRNAs, a summary is difficult to make. But, in this review we intend to do a summary of the current knowledge by focusing on miRNA as diagnostic, predictive and prognostic biomarker in CRC. Also, a short section about therapeutic role of miRNA in cancer will be described.

## 2. miRNAs and lincRNA Mechanism of Action

miRNAs act throught a post-transcriptional regulation that depends on the degree of complementarity between a miRNA and its target. Imperfectly base-pair with sequences in the 3′-UTR of target mRNAs and miRNA inhibit protein synthesis by either repressing translation or promoting mRNA deadenylation and degradation. On the other hand, when a miRNA and an mRNA exhibits perfect complementarity, the target mRNA is cleaved by RISC [24]. Imperfect base pairing between a miRNA and its target, as occurs with most mammalian miRNAs, leads to translational silencing of the target [25]. However, imperfectly complementarily miRNAs can also reduce the abundance of mRNAs [26].

Prediction of miRNA targets is one of the most important fields in miRNAs research, given that miRNAs exert their function by regulating target mRNAs. The specificity of miRNA–mRNA interaction is mainly conferred by the first eight nucleotides of a miRNA (known as seed sequence). However, the likelihood that a predicted target is a bona fide target is influenced not only by seed pairing but also by other factors such as the number of target sites, the context of surrounding sequence in mRNA [27], and the occlusion of target sites by RNA-binding proteins [28]. Currently, various algorithms have been developed for predicting miRNA target interactions. [29–39]. Traditionally, some major characteristics such as the hairpin-shaped stem loop structure, high evolutionary conservation, and high minimal folding free energy are important features used in the computational identification of miRNAs targets. These programs indicate that each miRNA potentially regulates hundreds of target mRNAs [40], and it seems plausible that most, if not all, mRNAs are post-transcriptionally regulated by miRNAs. However, the most important problem of these computational algorithms remains target over-prediction. Many targets predicted by *in silico* analyses are not confirmed as real targets in biological assay. Thus, the gold standard for miRNA target identification is the experimental demonstration that (*a*) a luciferase reporter fused to the 3′-UTR of the predicted target is repressed by overexpression of the miRNA and (*b*) this repression is abrogated by point mutation in the target sequence(s) in the 3′-UTR [41].

Very little is known about lincRNA biogenesis, in contrast with miRNAs, previous mechanisms of processing are not necessary for lincRNAs. These long non-coding RNA are exclusively intergenic and are marked by a chromatin signature indicative of transcription [5,13].

**Figure 1:**
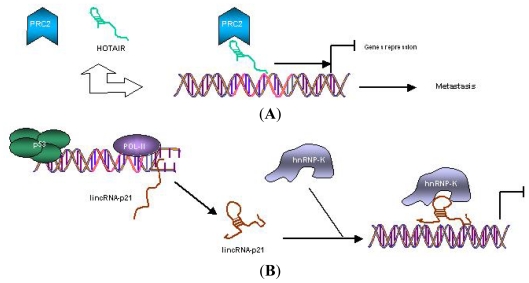
(**A**) Mechanisms of gene regulation by oncogenic lincRNA HOTAIR. HOTAIR recruits PRC2 to specific gene promoters, inducing gene repression that leads to tumor metastasis; (**B**) Mechanisms of gene regulation by tumor-suppressor lincRNA-p21, where its expression is directly induced by p53. Then, lincRNA-p21 specifically interacts with hnRNP-K for localization to gene promoters for repression [42].

Mechanisms of action of lincRNAs represented by two of them closely related to cancer developement: lincRNA-p21 described as tumor suppressive and lincRNA-HOTAIR as oncogenic (Figure 1 A and B, respectively).

## 3. miRNAs, lincRNA and Cancer

At the time of this writing it review, the latest version of the Sanger Institute miRBase (V16) [43], have been described 2108 human miRNA sequences [44].

Many evidences have indicated that miRNAs are involved in the tumorigenesis of many human cancers and, since their discovery, close to 3000 publications associate miRNAs to cancer, including over 700 reviews.

Cancer-associated genomic regions could be disrupted by chromosomal abnormalities, which include minimal regions of loss of heterozygosity, regions of amplification *etc*. These regions affected genes encoding miRNAs therefore connection between altered miRNA expression and cancer has been proved [45].

In this sense, the initial support for the involvement of miRNAs in cancer came from identification of miR-15a and miR-16a in the chromosome region 13q14, commonly deleted in human chronic lymphocytic leukemia (CLL). Expression analysis reported by Calin *et al*. [46] has indicated that miR-15 and miR-16 were either absent or down regulated in the 68% of CLL patients. It was later shown that miR-15a and miR-16-1 expression silenced the anti-apoptotic factor *BCL-2*, suggesting that their absence in CLL inhibit apoptosis by reactivation of *BCL-2* [47]. For the first time, it was showed that miRNAs have a potential role as tumor suppressor.

It has been predicted that miRNA regulate near 20% to 30% of genes [48]. Between these targets regulated by miRNA including genes involved in diverse biological processes and each of them probably controls hundreds of genes. Thus, altered levels of miRNA have been shown in different systems, such as oncogenesis and development. Thus, a large scale of miRNA expression profiling analyses reveals that mature miRNAs are globally down regulated in various tumors and profiling and characterization studies have proved that it could be used to create signature for many malignancies and classify human cancer [49–54]. Therefore miRNA may inhibit or promote tumor progression depending on mRNA target function. On the other hand, lncRNAs are also emerging as important regulators in pathways that involve oncogenes and tumor suppressors. Misregulation of antisense ncRNAs encoded by the opposite strand of protein-coding transcript partners affect the expression of the sense gene [55]. Epigenetic silencing of a tumor suppresor gene by the expression of the antisense ncRNA could lead to pathogenesis transformation when its regulation is disrupted. Chromatin-regulatory complexes are linked with the aberrant proliferation of cancer cells and around 20% of large intergenic ncRNA (lincRNAs) are associated with these chromatin-repressive complexes. Among them, polycomb repressive complex 2 (PRC2)’s ability to repress specific subset of target genes is affected when some lincRNAs are depleted [56].

Some lincRNAs modulate tumor suppressor pathways, like linRNA-p21, directly induced by p53 to play a critical role in the p53 transcriptional response. LincRNA-p21 functions as a gene repressor by interacting with hnRNP-K protein, allowing its localization to gene promoters to be repressed by p53 [57].

## 4. miRNA Expression in CRC

The central role of miRNAs in development and their direct effects in global gene regulation can indicate that miRNAs are important markers for human cancer. It has been suggested that the tumor miRNA profile may resemble that of their antecedent stem cells and thus reflect development lineage. In this sense, Lu *et al*. [58] analyze the expression levels of 217 miRNAs across 334 primary tumors, normal tissues and cell lines and their results showed that tumors display a miRNA expression profile reminiscent of that in the tissues from which they were derived. The miRNA profile was a better indicator of tissue lineage than was mRNA profile. Our understanding of miRNA function in mammals suggests that these molecules play a role in determination and/or maintenance of lineage during development. The alteration of miRNA expression in tumors samples might indicate the reduced differentiation that is a property in cancer.

Colon cancer is also associated with altered miRNA expression. Michael *et al*. [58] identified by cloning technique that the expression of two mature miRNA, miR-143 and miR-145, was consistently reduced at the adenomatous and cancer stages of colorectal cancer. Later miRNA serial analysis of gene expression (miRAGE) was utilized to compare expression levels of miRNAs in two primary colorectal adenocarcinomas with matched normal colonic epithelia. Cummins *et al*. [60] identified over 50 differentially expressed miRNAs, and miR-145 and miR-143 were in both studies significantly lower in tumoral cells compared to normal colonic cells. We examined the expression of 156 mature miRNA, consecutively, by Real-time PCR, in a panel of 16 CRC cell lines and 12 matched-pairs of tumoral and non-tumoral tissues from patients [61]. We identified a subset of 13 miRNAs differentially expressed in CRC cell lines as well as patients matched normal and tumoral tissues; among them miR-145 was also identified as down-regulated in CRC tissues. Moreover, the expression levels of miR-31 were higher in the tumor samples and CRC cell lines in comparison to the non-tumoral samples and were related to pathological stage, suggesting that this miRNA could contribute to both, the tumorogenesis and the acquisition of a more aggressive phenotype in CRC.

## 5. ncRNA as Regulator of Tumor Hallmarkers and Vogelstein’s Model of CRC Pathogenesis

**Figure 2:**
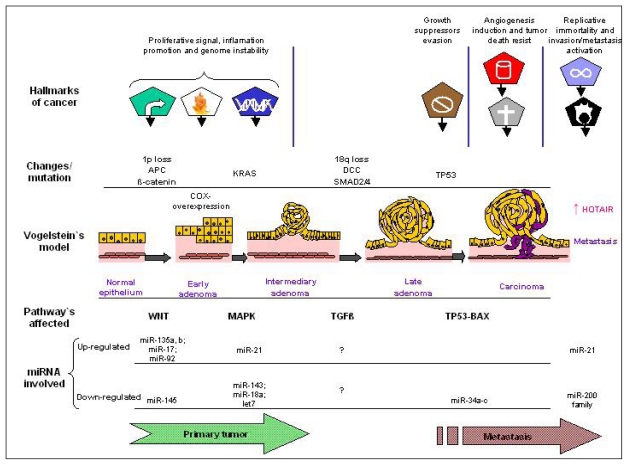
Connection between emerging hallmarks of each acquired capabilities necessary for tumor growth and progression [23] and, Vogelstein’s model of CRC pathogenesis [63]. MicroRNAs’ involved in each way and emerging linc-HOTAIR implication in metastasis features.

Many studies have highlighted the biological importance of miRNAs in CRC development, including genesis, progression and response to treatments. These studies have shown that many proteins involved in key signaling pathways of CRC, such as members of the Wnt/β-catenin and phosphatidylinositol-3-kinase (PI-3-K) pathways, KRAS, p53, extracellular matrix regulators, as well as epithelial-mesenchymal transition (EMT) transcription factors, are altered and seem to be affected by miRNA regulation in CRC [62]. These findings significantly extend Vogelstein’s model of CRC pathogenesis [63] and have shown the key role of miRNAs in CRC development. Carcinogenesis process, the so called “tumor hallmarkers” described by Hannahan and Weinberg [23], could be linked to this model in addition with miRNA role in this well know pathological process (summarized in Figure 2).

The Wnt/β-catenin pathway plays a central role in an early colorectal tumor development. Inactivation of the adenomatous polyposis coli (APC) gene is a major initiating event in colorectal carcinogenesis occurring in more than 60% of colorectal adenomas and carcinomas and leading to stimulation of the Wnt pathway via free β-catenin [63]. As shown by Nagel *et al*. [64], miRNAs represent a novel mechanism for APC regulation in CRC. miR-135a and miR-135b decrease translation of the APC transcript *in vitro*. Of note, miR-135a and miR-135b were also found to be upregulated *in vivo* in colorectal adenomas and carcinomas and correlated with low APC levels [22]. These observations indicate that alteration in the mir-135 family can be one of the early events in CRC’s molecular pathogenesis.

Another important key signaling pathway in CRC development is the EGFR pathway. Stimulation of EGFR and, subsequently, KRAS signaling lead to the activation of numerous signal transduction molecules initiating a cascade of downstream effectors that mediate tumor growth, survival, angiogenesis and metastasis in a broad spectrum of cancers, including CRC. KRAS oncogene has been reported to be a direct target of the let-7 miRNA family and of miR-143 [65]. More recently, miR-18a was observed to directly regulate KRAS but not N- and HRAS levels in the colon adenocarcinoma HT-29 cells [66]. Another central signaling downstream from EGFR and important in CRC development is the PI-3-K pathway. Studies based on microRNA arrays found a ubiquitous loss of miR-126 expression in CRC cell lines when compared to normal human colon epithelia and restoration of miR-126 expression result in a significant growth reduction [67]. The p85β regulatory subunit involved in stabilizing and propagating the PI-3-K signal was mechanistically proven to be a direct target of miR-126 [67].

EMT is the conversion of an epithelial cell into a mesenchymal cell. Morphologically, EMT is characterized by a decrease of E-cadherin, loss of cell adhesion, and increased cell motility leading to promotion of metastatic behavior of cancer cells (including CRC) [68]. The transcriptional repressor zinc-finger E-box binding homeobox 1 (ZEB1) is a crucial inducer of EMT in various human tumors, and it recently was shown to promote invasion and metastasis of tumor cells. The functional links to EMT comes from members of the miR-200 family (miR-200a, miR-200b, miR-200c, miR-141 and miR-429). ZEB1 directly suppresses transcription of miRNA-200 family members miR-141 and miR-200c, which strongly activate epithelial differentiation in pancreatic, colorectal and breast cancer cells [66].

Overexpressed cyclooxygenase-2 (COX-2) strongly contributes to the growth and invasiveness of tumor cells in patients with CRC [69]. It has been demonstrated that COX-2 overexpression depends upon various cellular pathways involving both transcriptional and post-transcriptional regulations. An inverse correlation was reported between COX-2 and miR-101 expression in CRC cell lines. It was demonstrated *in vitro* that the direct translational inhibition of COX-2 mRNA is mediated by miR-101. Moreover, this correlation was supported by data collected *ex vivo*, in which colon cancer tissues and liver metastases derived from CRC patients were analyzed. Impairment of miR-101 levels could represent one of the leading causes of COX-2 overexpression in CRC cells [69].

Macrophage migration inhibitory factor (MIF) is an innate cytokine, which plays a critical role in the host control of inflammation and immunity, and MIF could inhibit p53 tumor suppressor activity [68]. Interestingly, MIF has been found to play an important role in the colorectal carcinogenesis and hypoxia-induced apoptosis [71–73]. Recently, our group showed that MIF is a potential target of miR-451 [70]. Over-expression of miR-451 in gastric cancer and CRC cells resulted in reduction of cell proliferation, increased their susceptibility to radiotherapy, down-regulated expression of MIF at both mRNA and protein level. Furthermore, an inverse connection between miR-451 and MIF expression in biopsies of gastric tumors was observed, which suggested a role of miR-451 as a tumor suppressor. On the whole, miRNAs play a role in the signal pathway linking inflammation and tumorigenesis.

On the other hand, we showed recently the functional effects of miR-192/215 on cell cycle, likely due to their pleiotropic mechanism of action, reduce the 5-fluorouracil (5-FU) activity. 5-FU is an S-phase specific drug and previous studies underline the determinant role of cell cycle regulation in 5-FU sensitivity [74–76]. Moreover, we found that miR-192/215 increases p21 expression. In agreement with our results, CRC cell lines resistant to 5-FU had 3-fold higher p21 expression compared to sensitive cell lines [77].

Our findings also suggest a potential role of these miRNAs as biomarkers in CRC. According with recent studies [78,79], we found an anti-proliferative effect of miR-192/215 overexpression in CRC cell lines. Braun *et al*. [79] found that the expression of miRNA-192/215 was down-regulated in cancer tissue compared to normal mucosa. Moreover, two studies found a downregulation of miR-192 in CRC tissues when compared to the normal colon [80,81]. This evidence highlights the important role of miR-192 in CRC development and supports the idea that miR-192 might carry out a tumor suppression function. Moreover, cell cycle arrest in response to DNA damage is an important anti-tumorigenic mechanism. Several miRNAs were shown to play key regulatory roles in cell cycle progression. For example, miR-34a is induced in response to p53 activation and mediates G1 arrest by down-regulating multiple cell cycle–related transcripts. Braun *et al*. [79], using a direct pharmacologic activator of p53 identified two clusters of miRNAs comprising miR-192/215 regulated by p53. The same miRNAs were also up-regulated by DNA damage in a p53-dependent fashion like miR-34a [78], and in accordance with our results, activation of miR-192/215 induces cell cycle arrest, suggesting that these miRNAs operate in the p53 network. Furthermore, Georges *et al*. [78] define a downstream gene expression signature for miR-192/215 expression, which includes a number of transcripts that regulate G1 and G2 checkpoints. Of these transcripts, 18 transcripts are direct targets of miR-192/215; finally, the authors concluded that observed cell cycle arrest most likely results from a cooperative effect among the modulations of these genes by the miRNAs.

Regarding lincRNAs, a recent study reports association of HOTAIR expression and poor prognosis in CRC [82]. They measured Hotair expression by quantitative real time PCR in 100 colon cancer tissue samples matched normal tissue and report significant difference between them. When they considered only tumor samples and divide then between lower and higher Hotair expression, strong association was found between high expression and liver metastasis and poor patient prognosis. Moreover, cDNA microarrays from a specific subgroup of CRC samples obtained by laser micro dissection (LMD) suggested that Hotair expression induced genome-wide re-targeting of PRC2 in this CRC study group. Finally, *in vitro* studies confirmed invasion promotion by Hotair over-expression. Thus, the increase of undifferentiated cancer cells could be stimulated by Hotair expression changing the regulation of multiple genes by the loss of cooperation with PRC2 complex.

Altogether these studies underscore the key role of miRNAs and recent described lincRNA in cancer development, including CRC. To improve the knowledge of the roles of miRNAs in CRC pathogenetic pathways, functional effects of particular miRNAs have been successfully studied showing that epigenetic regulation by miRNAs and some lincRNA (as HOTAIR) in CRC is highly critical and complex (Figure 2).

## 6. miRNA and Chemoresistance

Comparison of the expression patterns of miRNAs and the potency patterns of 3089 chemical compounds has shown significant correlations, suggesting that miRNAs have a role in chemoresistance [83]. First, miRNAs expression was measured in 60 human cancer cell lines (the NCI-60) previously used to screen >100,000 compounds as potential anticancer drug agents. Correlations between microRNA expression and compound potencies suggested potentially relevant drug-microRNA pairs. Finally, three microRNAs known as oncomiRs, mir-21, let-7i, and mir-16, were transfected in three of NCI-60 cell lines and the effect of their expression on the potencies of a number of compounds with anticancer activity was tested showing a substantial role for microRNAs in anticancer drug response and suggesting a novel potential approach to the improvement of chemotherapy [84].

Resistance of cancer cells to chemotherapy continues to be a major clinical obstacle to the successful treatment of cancer [85]. At present, the anticancer drug resistance is considered as a multifactorial phenomenon involving several major mechanisms [86]. Causes of cancer specific drug resistance are currently believed to be linked to the random drug-induced mutational events (genetic hypothesis), to the drug-induced non-mutational alterations of gene function (epigenetic hypothesis), and, recently, to the drug-induced karyotypic changes [87–91]. Different pathway have been described as implicated in drug cancer resistance such as decreased uptake of water-soluble drugs, increased repair of DNA damage, reduced apoptosis, altered metabolism of drugs and increased energy-dependent efflux of chemotherapeutic drugs that diminish the ability of cytotoxic agents to kill cancer cell, changes in glutathione transferase, topoisomerase II and in microtubule related genes expression.

Cancer stem cells (CSC) are a small subpopulation of cells identified in a variety of tumors that are capable of self-renewal and differentiation. Deregulation of stem cell self-renewal is a likely requirement for the initiation and formation of cancer. Furthermore, cancer stem cells are proving to be a very likely cause of resistance to current cancer treatments, as well as relapse in cancer patients.

Genetic, epigenetic and the post-transcriptional regulation by miRNAs of genes involved in CSC maintenance, also contribute to drug resistance.

Ragusa *et al*. [92] found that miR-146b-3p and miR-486-5p were more abundant in mutant KRAS patients compared with wild-types suggesting that these miRNAs are involved in EGFR pathway. They also investigated miRNAs profiling in two human CRC cell lines, one sensitive and the other resistant to cetuximab (Caco-2 and HCT-116, respectively). Caco-2 and HCT-116 miRNAs profile was also studied after treatment with cetuximab. The authors suggested that the down-regulation of let-7b and let-7e and up-regulation of miR-17-3p were potential predictive markers of cetuximab resistance. However, until now, no clinical data confirms these findings.

Let-7g and miR-181b were also shown to be associated with chemosensitivity to S-1 (a pro-drug of 5-FU) based chemotherapy in colon cancer. The roles of let-7g and miR-181b in chemosensitivity are associated with their regulation of several genes such as RAS, cyclin D, C-MYC, E2F and cytochrome C [93]. These genes have been shown to be important for the transduction of cell signals, the control of cell cycle and chemosensitivity. However, the detailed molecular and cellular mechanisms of let-7g and miR-181b in mediating translational control will require further studies to elucidate the link of these miRNAs in chemosensitivity to fluoropyrimidine-based drugs in colon cancer.

MiR-34a was identified as one of the down-regulated miRNAs in human CRC 5-FU-resistant DLD-1 cells compared with those in the parental DLD-1 cells. miR-34a was also found down-regulated in drug resistant prostate cancer cells and ectopic miR-34a expression resulted in cell cycle arrest and growth inhibition and attenuated chemoresistance to the anticancer drug camptothecin [94].

Recently, Valeri *et al*. [95] demonstrated that miR-21 targets and down-regulates the core MMR recognition protein complex, human mutS homolog 2 (hMSH2) and 6 (hMSH6). Colorectal tumors that express a high level of miR-21 display reduced hMSH2 protein expression. MMR impairment appears to cause reduced incorporation of 5-FU metabolites into DNA, leading to reduced G2/M arrest and apoptosis after 5-FU treatment. Cells that overexpress miR-21 exhibit significantly reduced 5-FU-induced G2/M damage arrest and apoptosis that is characteristic of defects in the core MMR component. These results were confirmed in xenograft studies demonstrating that miR-21 overexpression dramatically reduces the therapeutic efficacy of 5-FU. These studies suggest that the downregulation of the MMR mutator gene associated with miR-21 overexpression may be another important clinical indicator of therapeutic efficacy of 5-FU in CRC.

Another study determined that 5-FU and oxaliplatin (L-OHP) down-regulated the expression of miR-197, miR-191, miR-92a, miR-93, miR-222 and miR-826 in HCT-8 and HCT-116 colon cancer cells [96]. These results indicate that 5-FU and L-OHP mechanism of action could rely in part on their influence on the down-regulated miRNA expression providing novel molecular markers.

Svoboda *et al*. [97] reported that median levels of miR-125b and miR-137 were upregulated in rectal cancer patients after a short-course of capecitabine-based chemoradiotherapy, and higher induction of miR-125b and miR-137 were associated with worse response to the treatment. For the first time, in this clinical study the miRNAs modification during therapy in patients with rectal cancer undergoing chemoradiotherapy with capecitabine was investigated. Importantly, while a number of these miRNAs showed distinct variation two weeks after starting therapy, showing profound inter-tumoral variability, miR-125b and miR-137 demonstrated a significant induction and similar expression trends. The increased levels of both miRNAs correlated with minor response to therapy and with higher, post-surgery, tumor stage suggesting that higher induced levels of miR-125b and miR-137 might be associated with worse response to radiotherapy with capecitabine.

Many efforts have been exerted in analyzing the role of miRNAs in the development of drug resistance in a variety of malignancies, including CRC. Several research groups have shown that the expressions of miRNAs in chemoresistant cancer cells and their parental chemosensitive ones are different. The molecular targets and mechanisms of chemosensitivity and chemoresistance are also successfully studies. These results suggest a great potential for miRNAs as predictive biomarkers and chemotherapy modulators.

## 7. SNPs and miRNA

Although single-nucleotide polymorphisms (SNPs) in miRNA regions are rare and considered unlikely to be functionally important [98] nucleotide variations within the seed sequence of the miRNA or on primary (pri) and precursor (pre) miRNAs might affect its processing and ultimately lead to modification of its expression [99]. Therefore, it is plausible that SNPs in miRNA biogenesis machinery genes and miRNA-containing genomic regions may affect thousands of target mRNAs, these or their targets might represent ideal candidate biomarkers implicated in cancer development, prognosis and prediction of cancer patients clinical outcome.

In this sense, SNPs in miRNA-biogenesis genes might affect the expression of mature miRNAs and consequently miRNA-mediated regulation within the cell.

Recently, we developed a study of SNPs involved in miRNA biogenesis in a group of CRC patients homogenously treated with 5-Fluorouracil plus Irinotecan to investigate whether these polymorphisms may influence the outcome of metastatic colorectal cancer (mCRC) patients [100]. We found that the SNP rs7372209 in miR-26-a-1 was associated with overall response rate and time to progression (ORR and TTP, respectively). Allele C appears to be a favorable factor, as was confirmed comparing the median TTP of CC+CT genotypes to the homozygote variant (TT). Furthermore, the SNP rs1834306 in the 5′-UTR region of pri-miR-100 and the SNP rs11077 in the XPO5 gene (Xportin 5) were also found to be associated with TTP and DCR (disease control rate), respectively.

Interestingly, when we performed the *in silico* analysis for putative target genes of these miRNAs, we found that some of them have been implicated in colon tumorigenesis and clinical outcome.

On the other hand, the first epidemiological study showing an association between microRNA-binding SNP sequences and cancer risk was performed by Landi and co-workers [101] through a case-control study that examined the association of eight polymorphisms within microRNA-binding sites with the risk of sporadic CRC, founding that the variant alleles of *CD86* and *INSR* genes were strongly associated with the risk of CRC.

In a more recent study of early stage CRC patients, a SNP in a *let-7* miRNA complementary site (LCS6) in the *KRAS* 3′untranslated region (*KRAS-LCS6*) genotype combined with *KRAS* mutations seemed to affect survival in metastatic patients [102]. Although this result could be considered interesting concerning patients’ outcome, it also merits to be validated as a prognostic biomarker and to be considered in therapy-decision-making.

## 8. miRNAs as Predictive and Prognostic Biomarkers in CRC

A number of studies based on expression profiling have proven that there are significant changes of miRNA expression levels in CRC tissue in comparison to normal colorectal epithelium. Moreover, comparing miRNAs expression in groups of patients with different clinical outcome have been identified several miRNAs with prognostic value.

Recent studies on miRNAs have shown that the expression levels of different miRNAs, such as miR-21, miR-320, miR-498, miR-106a and miR-200c correlate with the probability of recurrence-free survival in CRC stage II-III. Schetter *et al*. [80] compared miRNA expression patterns in stage II colonic adenocarcinoma and adjacent normal tissue using a test set and two validation cohorts. In one of the cohorts, a high tumor to normal expression ratio of miR-20a, miR-21, miR-106a, miR-181b and miR-203 was associated with poor survival. In the second validation set, including stage III, survival analysis showed that higher miR-21 expression predicted poorer survival in treated colonic cancer and poor responsiveness to adjuvant chemotherapy.

Similar associations have been found by other research group. Schepeler and colleagues [103] showed that miR-320 or miR-498 expression was significantly associated with progression-free survival in stage II CRC. These miRNAs were found to be independent predictors of recurrence-free survival when stratified for age, sex, tumor stage, differentiation and histological grade.

Another study found that miR-21 and miR-31 were significantly up-regulated, and miR-143 and miR-145 down-regulated in tumors compared with the normal counterparts. High expression of miR-21 was associated with lymph node positivity and distant metastases and tumors >50 mm in maximal tumor diameter were related to lower expression of miR-143 and miR-145 [104,105]. The expression level of miR-31 was correlated with the stage of CRC [59]. Further study confirmed this result showing that miR-31 expression was positively related to advanced TNM stage and deeper invasion of tumors, suggesting over-expression of miR-31 might be involved in the development and progression of CRC [104]. In addition, miR-18a was found to imply a poorer clinical prognosis [106].

Some groups also demonstrated that miRNAs expression is associated with microsatellite status in CRC. Twenty-three colon cancer samples characterized by microsatellite stability (MSS), and 16 by high MSI were studied for genome-wide expression of miRNA and mRNA. Eight miRNAs was found correctly distinguish MSI-H *versus* MSS colon cancer samples based on combined miRNA and mRNA gene expression [107].

In Table 1 we summarized some of the most important miRNAs found associated to CRC clinic-pathological features and clinic outcome.

Overall, these findings suggest that a growing number of miRNAs appear on the potential biomarker candidates list for CRC. An important advantage of using miRNAs for biomarker identification is the stability of the miRNAs in archival specimens. This is a key factor in large-scale retrospective biomarker discovery studies. The different length of formalin fixation time and paraffin embedding has little impact on miRNA stability. This is evidently explained both by the shortness of the molecules as well as protection from degradation by intimate RNA-protein interactions. Other factors may be that miRNA sequences could have evolved to elude RNA nucleases. Whereas mRNA tends to be labile in fixed and/or embedded tissue [109], a number of studies have shown robust correlations between miRNA profiling results in fresh, *versus* in formalin fixed paraffin-embedded (FFPE) tissue [110,111]. This ensures that miRNA biomarker discovery using archival FFPE specimens with a large sample size is feasible. miRNAs were also found to be stable in blood and body fluids, which has expanded the biomarker discovery sample types [112]. Moreover, recent findings have proven that, due to miRNAs stability, stool miRNA detection might be used as a new diagnostic biomarker for early CRC detection [113].

Under disease conditions such as cancer, miRNAs entered circulation predominantly from tumor cells [114]. Recent studies indicated that tumor-derived miRNAs are resistant to endogenous ribonuclease activity detectable in plasma/serum. Thus, body fluids were also found to be a stable sample source for this study.

Studies developed three years ago showed potential use of miRNAs as novel candidates of diagnostic and/or prognostic “circulating marker” of several cancer types [108,112,114–116]. Its remarkable stable form and the possibility of extraction from serum samples make them reproducible and consistent among individuals [117].

Although the analysis of circulating miRNAs from serum over plasma or even whole blood are promising for systemic miRNA study, their future application for diagnosing or prognosis of human cancers is questionable and there is no consensus on the optimal circulating medium or isolation technique from which to quantify these miRNAs. Accumulating evidence supports *miR-16* as a potentially ideal normalizing miRNA gene. This is abundantly expressed in blood and many solid tissues have been shown to reveal expression in tumor and normal specimens by several authors [115,117]. Despite miRNAs frequently being chosen for blood-based qRT-PCR analysis, this is a contentious yet critical issue and the ideal normalization control for this has not yet received a consensus [118].

## 9. miRNAs as “One Plus” Potential Cancer Therapeutic Targets

The fact that miRNA dysregulation in cancer has a pathogenic effect provides the rationale for using miRNAs as potential therapeutic targets in cancer.

Typically, miRNAs that serve as oncogenes are present at high levels in tumors, which inhibit the transcription of genes encoding tumor suppressors. Conversely, tumor suppressor miRNAs are present at low levels, resulting in the overexpression of transcripts encoded by oncogenes.

For miRNAs with oncogenic capabilities, potential therapies include anti-miRNA oligonucleotides, microRNA sponges, miRNA masking, and as small molecule inhibitors. For tumor suppressor miRNAs, restoring suppressor miRNAs by forced expression of those miRNAs may be a useful strategy. In several tumor types with global decreasing miRNA biogenesis, approaches to enhance miRNA biogenesis processing can also represent a valid therapeutic action.

The binding of miRNAs to their mRNA binding targets are simply and elegantly governed by the rules of Watson–Crick base pairing. Therefore, an obvious inhibitory molecule of miRNA is anti-miRNA oligonucleotides (AMOs) which blocks the interactions between miRNA and its target mRNAs by competition. AMOs are chemically modified in a variety of ways, such as with locked nucleic acid (LNA) [119], 2′-*O*-methyl- [120], and 2′-*O*-methoxyethyl-modified (2′-MOE) oligonucleotides [121], to improve their stability and to reduce their degradation.

A critical role of mir-21 in cell proliferation by down-regulating the tumor suppressor genes Tpm1 and PTEN has been suggested in [122]. In this way, mir-21 represents one of the first examples of inhibiting cancer development by down-regulating its oncogenic miRNA activity. Using a xenograft carcinoma model, Si *et al*. [123] injected MCF-7 cells transiently transfected with 2-*O*-methyl oligonucleotides complementary to mir-21 and found that tumors derived from MCF-7 cells transfected with anti-mir-21 were 50% smaller in size than control MCF-7 tumor.

Small molecule inhibitors against specific miRNAs have also been investigated. Gumireddy *et al*. identified azobenzene as a specific and efficient inhibitor of biogenesis of mir-21 from a screening. Such specific inhibitors of the miRNA pathway provide, not only unique tools for the investigation of miRNA function, but also promising reagents to boost patient response to existing chemotherapies or stand-alone cancer drugs [124]. However, the *in vivo* efficacy of such small-molecule inhibitors needs to be explored.

Other inhibitory molecules correspond to microRNA sponge, a synthetic mRNA containing multiple binding sites for an endogenous miRNA, therefore preventing the interaction between miRNA and its endogenous targets. Thus, to increase the efficacy of AMOs, Ebert *et al*. [125] engineered such molecules by inserting a bulge between the microRNA binding sites at the position normally cleaved by Argonaute 2, enabling stable association of microRNA sponges with microribonucleoprotein complexes loaded with the corresponding micro-RNA. In *in vitro* experiments, these “sponges” strongly derepressed miRNA targets, but their efficacy *in vivo* still needs to be evaluated.

Consider that each miRNA may regulate hundreds of genes, and each gene can be regulated by multiple miRNAs, the possibility that miRNAs can affect multiple targets with a single hit, represents an advantage of miRNAs over siRNA/shRNAs (small interfering RNA and short hairpin RNA, respectively). However, similar to siRNA/shRNAs, miRNAs may also interact with multiple targets and this effect is seen as a disadvantage, because off-target effects are largely unpredictable. Similar to endogenous miRNA, the action of AMOs is sequence-specific but not gene-specific. Thus, AMOs may elicit off-target side effects and unwanted toxicity. Xiao *et al*. [126] designed alternative strategy called “miR-Mask” which refers to a sequence with perfect complementarity to the binding site for an endogenous miRNA in the target gene, which can form a duplex with the target mRNA with higher affinity, therefore blocking the access of endogenous miRNA to its binding site without the potential side effects of mRNA degradation by AMOs.

On the other hand, restoring expression of miRNAs with tumor suppressive properties might be a successful strategy and can be achieved by transfecting miRNA mimics either directly or through vectors. For example, to re-introduce miR-34 and its tumor suppressor capabilities, transfection with miR-34 mimics into cancer cells was shown to block the cell cycle in the G1 phase, significantly increasing activation of caspase-3, and knocked down its downfield targets of bcl-2, Notch, and HMGA2 [127]. The miRNA mimic, is therefore restored miR-34 with its tumor suppressor potential; however, the transfection of the miR-34 mimics can only last a couple of days and the long-term biological effects were not observed very effectively. To overcome this dilemma, the cancer cells were infected with a lentivirus that expressed miR-34a. This generated stable cells expressing miR-34a. The lentiviral miR-34a was found to be able to inhibit cancer cell growth and tumorsphere formation [127].

Given the role of miRNAs deregulation in cancer stem cells, restoring the expression of miRNAs specifically down-regulated in CSC has been developed. In this sense, a lentiviral system restored the tumor suppressor effect of miR-34 in pancreatic cancer stem cells [128]. Therefore, miRNA mimics and lentiviral miRNAs show great potential in restoring tumor suppressor miRNAs to correct the dysregulation of critical genes in cancer, including cancer stem cells.

However, from a clinical/translational research point of view, for the miRNA-based therapeutics to be effective, the efficient and functional delivery of miRNA mimics and/or antagonists to tumor remains a great challenge. In this sense, viral and non-viral delivery systems have been developed.

Viral vector-directed methods show high gene transfer efficiency but are deficient in several areas. The limitations of a viral approach are related to their lack of tumor targeting and to residual viral elements that can be immunogenic, cytopathic, or recombinogenic. On the other hand, non-viral gene transfer vectors could circumvent some of the problems associated with viral vectors. Progress has been made toward developing non-viral, pharmaceutical formulations of gene therapeutics for *in vivo* human therapy, particularly cationic liposome mediated gene transfer systems. One disadvantage of cationic liposomes is that they lack tumor specificity and have relatively low transfection efficiencies as compared to viral vectors. However, this can be dramatically increased when the lipoplexes bear a ligand recognized by a cell surface receptor [129,130]. Receptor mediated endocytosis represents a highly efficient internalization pathway in eukaryotic cells. Non-Viral Delivery: More recently, a method of miRNA delivery using polyethylenimine (PEI)-mediated delivery of unmodified miRNAs was reported. After systemic or local application of low molecular weight PEI/miRNA complexes, intact miR-145 and miR-33 molecules were delivered into mouse xenograft tumors where they caused profound anti-tumor effects. miR-145 delivery reduced tumor proliferation and increased apoptosis, with concomitant repression of c-Myc and ERK5 as novel regulatory target of miR-145. Similarly, systemic injection of PEI-complexed miR-33a was validated as a novel therapeutic targeting method for Pim-1, with anti-tumor effects comparable to PEI/siRNA-mediated direct *in vivo* knockdown of Pim-1 in the model. These findings demonstrate that chemically unmodified miRNAs complexed with PEI can be used in an efficient and biocompatible strategy of miRNA replacement therapy, as illustrated by efficacious delivery of PEI/miR-145 and PEI/miR-33a complexes in colon carcinoma [131].

Therapeutic delivery of synthetic mi-RNA, using a neutral lipid emulsion (NLE), exhibited tumor-inhibitory effects of *let-7* and miR-34 formulations in an autochthonous transgenic mouse model of lung cancer. This model is based on oncogenic *KRAS^G12D^* that is expressed from a Cre recombinase dependent allele (*LSL-KRAS G12D*) containing native 5′ and 3′ untranslated regions. The results show that systemic delivery of miR-34a mimics can effectively cause reduction of advanced lung tumors in a *Kras* activated NSCLC mouse model through inhibition of proliferation and induction of apoptosis. Where, *let-7* treated tumors showed reduced proliferation. Consider that these miRNA might affect distinct cancer pathways and frequent downregulation in human lung tumors, then the combination of both *let-7* and miR-34a might yield superior therapeutic effects [132]. Besides, NLE delivery systems are less toxic than those containing cationic lipids and have more advantages, such as: non-accumulation of miRNA in the liver, being less likely to form aggregates in biofluids or be taken up by scavenging macrophages [133].

Through pre-clinical assays, it was established that small-interfering RNA (siRNA) molecules could be delivered to prostate tumor cells in a bone metastatic site using an atelocollagen delivery method. Further, reduced expression of cancer-related genes in miR-16-transfected prostate cancer cells was analyzed and verified that genes associated with cell-cycle progression were mostly down regulated by synthetic miR-16. In this study, atelocollagen facilitates the accumulation of enough synthetic miRNA in the cancer cells. These results suggest the therapeutic potency of miR-16 in bone metastatic prostate cancer [134]; although some discrepancies between other miRNA and vector types’ influence can be implicated in growth inhibition [135].

Another more recent pre-clinical study, identified and validated miR-34a as a novel therapeutic agent against prostate cancer stem cells (CSCs) by direct functional targeting of CD44 cells [136]. Where, overexpression of miR-34a exerts a negative effect over CD44^+^ prostate cancer cells acting as potent antitumor and antimetastasis in this cancer type. The most up-to-date molecular diagnostic cancer research findings [137], point to miR-34a expression and p53 status in lung cancer patients as the most promising prognostic marker.

Finally, with all the efforts and advances made in developing miRNA-mediated therapy, two major hurdles still remain. The first is to maintain target specificity. It is especially challenging since off-target gene silencing only requires partial complementary binding between miRNA and protein-coding transcripts. Therefore, it is important to evaluate the effect of a specific miRNA-mediated therapy on a proteome-wide scale to prevent unwanted gene alteration.

The second hurdle is to achieve high therapeutic efficiency. Two factors that can limit miRNA therapeutic efficiency is the amplitude of target gene modulation and the number of cells that can be targeted. To address the first limitation, one should optimize target gene selection as well as therapeutic molecule design. The second limitation comes from delivery efficiency.

Altogether these studies have indicated that miRNAs can serve as novel therapeutic targets for cancer. However, many challenges remain open. Although this kind of therapy is still a long way from fruition, as many issues need to be addressed before it can be successfully pursued, there is no doubt that we are at the dawn of a better understanding of key regulatory mechanisms in human gene regulation.

## 10. Summary

Differential expression of miRNA in cancer could be useful in the diagnosis and may distinguish between malignant and reactive lesions [138]. Moreover, given that the complex mechanism of gene regulation by microRNAs is profoundly influenced by variations in gene sequences (polymorphisms) of the target sites, it may indicate that miRNA expression could be influenced not only by tumor types, but also by individual variability related to ethnic geographical region.

On the other hand, in considering critical roles for chromatin-modifying complexes in the establishment and maintenance of cells pluripotency, the possibility of lincRNAs interactions with these complexes could impart target specificity. LincRNAs (as HOTAIR) could represent an additional layer of complexity in the networks controlling cellular differentiation.

Lastly, miRNA-based cancer therapy is a very interesting field of investigation that offers the appeal of targeting multiple gene networks controlled by a single miRNA such as described by Henry JC reviews [139].

## Figures and Tables

**Table 1:** Relevant miRNAs found deregulated in CRC expression profiling.

miRNA	Dysregulation	Clinical-related phenotypes	Reference
Let-7g	Upregulated in CRC	Higher level associated with poor S-1 response	[93]
miR-18a	Upregulated in CRC	Higher level associated with poor overall survival	[106]
miR-21	Upregulated in adenoma, CRC, and liver metastasis tissue	Higher level associated with lymph node positivity, metastasis; poor survival, poor therapeutic outcomes, rapid recurrence; shorter disease-free interval	[80,105]
miR-31	Upregulated in CRC	Higher level associated with higher TNM stages and local invasion	[61,105,108]
miR-106a	Upregulated in colon cancer	Higher level associated with longer disease-free survival and overall survival	[80]
miR-143	Downregulated in colon cancer and liver metastasis tissue	Lower level associated with larger tumor size and longer disease-free interval	[105,106,108]
miR-145	Downregulated in CRC	Lower level associated with larger tumor size; related with tumor location	[105,106,108]
miR-181b	Upregulated in CRC	Higher level associated with poor S-1 response	[93]
miR-320	Downregulated in MSS tumor	Lower level associated with shorter progression-free survival	[103]
miR-498	Downregulated in MSS tumor	Lower level associated with shorter progression-free survival	[103]
